# A Method for Characterising the Influence of Casting Parameters on the Metallurgical Bonding of Copper and Steel Bimetals

**DOI:** 10.3390/ma14206223

**Published:** 2021-10-19

**Authors:** Simon Kammerloher, Julika Hoyer, Philipp Lechner, Tim Mittler, Wolfram Volk

**Affiliations:** Chair of Metal Forming and Casting, Technical University of Munich, 85748 Garching, Germany; julika.hoyer@utg.de (J.H.); philipp.lechner@utg.de (P.L.); mittler.tim@t-online.de (T.M.); wolfram.volk@utg.de (W.V.)

**Keywords:** copper, steel, composite casting, bimetal, casting parameter

## Abstract

Traditional casting technology offers two mayor drawbacks towards research activities. On the one hand, time and resources needed for every casting are rather high. The mould has to be able to withstand the high temperatures introduced by the melt and provide cooling for the cast part. Preparation and installation of measuring equipment therefore takes time. Additionally, due to the high mass of the mould when compared to the cast part, parameter variations are rather limited in their resulting effect on the temperature-time profile being one of the most prominent factors regarding cast quality. Especially when pouring by hand, variations in casting times and rates superimpose effects created intentionally. Therefore, a different process was advanced and evaluated, allowing to minimise some of the drawbacks mentioned before. The key idea is to drastically reduce casting size to the dimensions of one specimen and to apply a highly automated production route. As such, a mirror furnace was modified as to allow the processing of melt. Due to the specimens size, an adaption of mechanical testing equipment was performed and evaluated. As an example, copper-iron bimetal specimens were examined by light microscopy, micro hardness testing, nanoindentation as well as tensile and torsion testing. As the results were consistent, the newly introduced method can be applied successfully in casting research. This allows for highly reproducible results, reducing the uncertainty of temperature measurements of a specimen due to the distance between them. The possibility of separating influencing variables like maximum temperature and cooling rate allows an analysis of the casting process, which would require different moulds to do so in traditional casting methods. The next steps will be directed at a broader variety of metals processed and at a direct comparison between the new process route and traditional casting technology.

## 1. Introduction

### 1.1. Compound Casting

The trend towards increased performance and efficiency of metallic components results in materials being stressed to their limits. Additionally, a material is chosen typically to suffice all requirements, rather than to fulfil one to the maximum. Therefore, monolithic components represent a compromise regarding performance, which could be solved by using the right material at the right location [1].

The connection between those different materials could be either force or form fit or feature a metallurgical bonding and is created by simultaneous or consecutive casting of two melts, or by casting a melt onto an metallic insert [2].

According to Ißleib, Friedel and Lubojanski, a precondition for the formation of a metallurgical bonding is contact on an atomic scale, whereas a cohesive bond requires solubility of both partners. They propose the following mechanisms during compound formation [3]:

- melting, crystallisation and cooling processes developing stresses;
- reciprocal diffusion of one cast material into the other, grain boundaries being favourable locations;
- polymorphic transformation; and
- structural transformation: dissolution and precipitation processes forming solid solutions and/or intermetallic phases.

The authors stress the complex interactions of the above mentioned phenomena, being heavily influenced by the interfaces thermal conditions. The resulting interface temperature is dependent on thermophysical properties of both materials, as well as casting and geometric parameters [3].

As such, the possibility to influence the interfaces thermal conditions within any given casting setup is rather limited. An approach provided by Mittler [4] was to adapt a mirror furnace, previously used for heat treatment. This allowed to set and hold interface temperature of a bimetal specimen within a graphite crucible freely. As a result, the separation of different mechanics of compound forming due to a variation of process parameters is possible, beyond what was feasible in casting [4].

### 1.2. Copper-Iron Compounds

Compounds featuring a copper and an iron base metal find application, e.g., in automotive industry and machinery as bearings [5] and in electrical industry as parts of plugs and switches [6]. The coppers antimicrobial effect provides for application possibilities as medical equipment or as parts of heat exchangers [7,8].

Copper-iron compounds are produced in several ways in industry and research. Steel cylinder blocks of piston pumps are clad with a bronze coating to reduce friction by melting a copper alloy within the steel part [9].

Roll cladding is a widely used technology for the production of strip-shaped hybrid semi-finished products. The bonding at the interface is induced by friction and pressure welding processes [10,11,12]. The necessary annealing steps between rolling also promote interdiffusion and forming of solid solutions and/or intermetallic phases [6,13].

In cast cladding a liquid phase is poured onto a strip-shaped base metal. Material combinations are, for example, copper-steel [14] and aluminium-steel [15]. Two phased wires, featuring a steel core and a copper outer layer, are produced by dipforming [16]. Another way to produce strip-shaped semi-finished copper-iron compounds is by explosive cladding. The bond is created by the explosions pressure [17]. The interface shows a nonuniformly shaped wave, thus the compound exhibits anisotropic behaviour. Diffusion lengths are very limited due to very short process times [18,19].

The bonding strength of diffusion-welded copper (CuAlBe)-stainless steel (1Cr18Ni9Ti) hybrids is increased by an intermediate layer of nickel. Microstructural analysis of the bonding zone shows that at low joining temperatures aluminium atoms react with nickel and iron to form AlNi and Fe ${}_{3}$Al. At higher temperatures, Cu atoms diffuse into the nickel layer and the Kirkendall effect occurs. This negatively influences bonding strength, due to leaving voids within the copper from where the atoms migrated into the steel. As such the volume of the steel part increases while the volume of the copper part decreases [20].

Interface investigations of cast aluminium-bronze-stainless steel hybrids show the formation of brittle intermetallic phases (AlCrFe_2_, Al_4_Cu_9_, AlNi_3_). The layer thickness of the intermetallic phases between the monolithic joining partners are ~0.15 mm thick [21]. In the academic field, developments of multilayer bearings made of steel, tin and copper were also carried out. This resulted in the intermetallic phases Cu_6_Sn_5_ and FeSn_2_ [22].

Under casting conditions, the solubility of copper in iron is 1–2 m-%. Alloying elements such as nickel, zinc, tin and aluminium are therefore necessary for the formation of intermetallic phases between copper and iron materials [23].

### 1.3. Research Goal

The aim of the current research is to refine and evaluate the approach introduced by Mittler [4] regarding a miniaturised casting setup. This allows for a wider range of possible temperature-time profiles within the casting material as would be possible to obtain in any given setup involving a classic mould. Once the heat capacities are defined, the temperature in such a setup can be varied only in a limited amount by preheating and cooling the mould and by different levels of overheating the melt. Therefore, some effects are hardly distinguishable, especially when overlain with process variations. The aim of this paper is to provide a highly controllable research setup, as to minimise these process variations. Furthermore, ways of specimen evaluation, especially regarding mechanical properties at small specimen sizes are investigated regarding the consistency of that data.

## 2. Materials and Methods

### 2.1. Specimen Preparation

For compound creation, the metals Cu-ETP and structural grade carbon steel were used. Their corresponding chemical composition is shown in Table 1.

The specimens were cut from rods with a diameter of 4 mm and ground to a length of 5 mm on a 600 grit abrasive paper. Thus, a uniform surface roughness Ra of approximately 0.4 μm for the steel and 0.5 μm for the copper specimens was achieved, leaving its influence on wetting and diffusion to a minimum. One in six Fe specimens was fitted with a hole reaching close to the opposite face. Prior to heating, the specimens were degreased in an ultrasonic bath, filled with ethanol. The steel specimens were coated on their circumference with HeBoCoat^®^ (Lauben, Germany) boron-nitride coating to prevent reactions with the graphite crucible, while leaving the face to be in contact with the copper specimen blank. To achieve a more uniform bonding area, the steel specimen was placed on top of the copper specimen in the crucible. A thermocouple was fed through the specimen holder and either installed into the hole or rested on the top face of the steel specimen. Then the crucible was screwed onto the specimen holder and both were inserted into the furnace chamber.

### 2.2. Mirror Furnace

**Figure 1:**
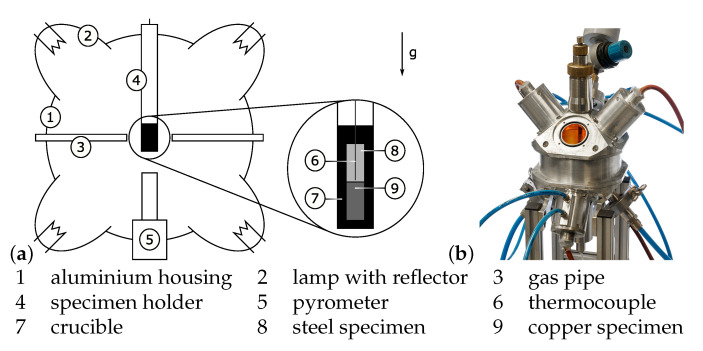
Mirror furnace (**a**) diagram with detail of crucible, and (**b**) the furnace.

The Cu-Fe compound was created in a fully automated furnace as depicted in Figure 1. The setup consists of a mirror furnace, essentially an aluminium ball with four halogen lamps attached to it. The lamps are mounted in concave mirrors, focusing their light onto the crucible at the centre of the chamber. Up to 0.8 kW can be set on the lamps, resulting in heating ramps of up to 60 °C/s. Prior to an experiment, the furnace is flooded with argon gas to protect the inside from oxidation. The gas is also used in quenching of the specimen, as the pipes are directed at the crucible. Temperatures are recorded both of the crucibles bottom surface by a pyrometer and of the Cu-Fe interface area by a thermocouple.

The power sources feeding the lamps as well as the argon-valve are controlled by a Labview program. Previous to an experiment, the specimens target temperature and its dwell time are specified. After flooding the furnace with argon, a PID-controller sets the desired lamps current in such a way, that a fast response of the controller is coupled with minimum overshoot. Upon reaching the final hold time, the lamps are turned off and the specimen is left to cool to room temperature. The lamps current, both temperatures and a logical indicator relating to argon-flow are stored at a set frequency.

The following experiments, which are used to create the specimens for mechanical testing feature Fe-specimen without a hole. As such, no relevant temperature data can be derived from the thermocouple. Hence those experiments are controlled as follows: Data from the first run are read for each time step to reproduce every action concerning argon and lamps. To compensate for differences in the experimental runs, e.g., a deterioration of the halogen-lamps, the crucibles bottom surfaces temperature is compared to that read from the file. That difference is fed into a PI-controller which regulates the lamps current to keep the difference at zero. A final run is conducted, yet again with a thermocouple installed into the steel specimen, but controlled according to the second version. Those runs act as validation for negligible differences in temperature-time profiles for the series.

### 2.3. Testing

**Figure 2:**
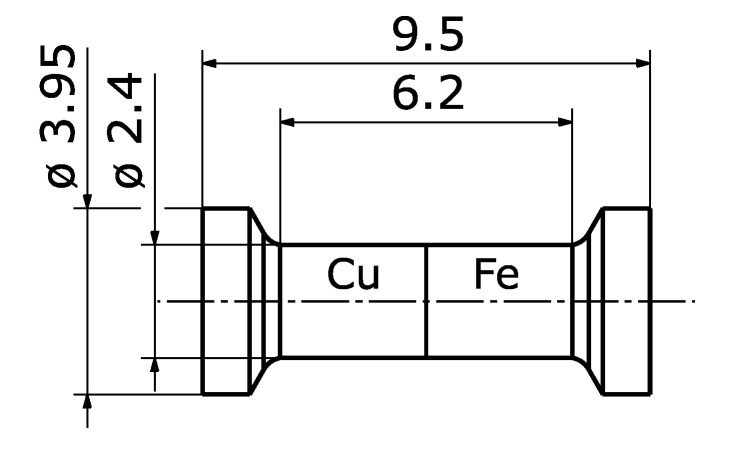
Mechanical testing specimen (values in mm).

Tensile tests were performed, using an universal testing machine of the type BT1-FB020TN.D30 by ZwickRoell GmbH, Ulm, Germany. The load cell works up to 20 kN and satisfies precision requirements of class 0.5, according to DIN EN ISO 7500-1 [24]. The tests were performed on the basis of [25], though with a deviation in shape due to the limited size of the specimens. The as-cast specimens were machined to featured form-fit as shown in Figure 2. The testing was performed with an uniform traverse speed of 2 mm/min until a preload of 50 N was reached. Afterwards, the speed was reduced to 1 mm/min. The test ended, once the load decreased to 50% of maximum load.

**Figure 3:**
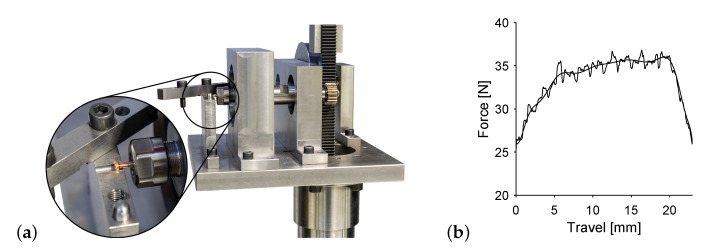
Torsion testing (**a**) test stand and (**b**) exemplary force-travel data.

Torsion testing was performed on the same universal testing machine as the tensile tests and used the same specimen shape as shown in Figure 2. The translational movement of the tensile testing device, as shown in Figure 3a, is transformed into a rotational movement acting on the specimen via gear rod and wheel. The specimens steel side is connected to the axle in a chuck, the copper side features two parallel flat surfaces on opposite sides to clamp the specimen, stopping rotation. Therefore, the tensile testing devices speed and force are connected to angular velocity and torque of the specimen via the cogwheels pitch circle diameter. Figure 3b shows an exemplary force over travel curve. Both raw data and smoothing are displayed, where the smoothing is done by fitting a Fourier function of 8th degree.

One specimen of each temperature-time level was embedded and ground down to its longitudinal centre plane. That surface was then polished as to obtain micrographs of both the etched and unetched state. The images were taken with the camera system AxioCam MRc 5, being part of the metallographic microscope Axioplan 2 by Carl Zeiss MicroImaging GmbH, Göttingen, Germany.

Microhardness was detected on the polished surface of the specimen using the testing apparatus was AHM43 by Leco Corporation, St. Joseph, MI, USA. The indents were arranged in a line parallel to the longitudinal centre. As such, hardness was measured depending on the distance to the interface of steel and copper. The measuring load was 0.05 N.

In order to gain further insight into hardness distribution close to the interface, a grid-like array of measurements were performed, reaching from the Fe into the Cu, using the nanoindenter NanoTest Vantage - Plattform 4 by Micro Materials, Wrexham, UK. A three-sided Berkovich tip was used and the indent was performed until a depth of 500 nm was reached with a dwell time of 20 s.

## 3. Results and Discussion

Specimen maximum temperature varied from 1090 to 1150 °C in steps of 10 °C. The cooling phase either started immediately after reaching maximum temperature (0), 10 or 30 s thereafter. As such, 21 different temperature-time variations were investigated, each producing 12 specimens.

Bonding occurs as soon as the copper specimen melts at the contact zone. When given more time, even below solidus temperature bonding could be observed. Any bonded specimens showed no immediately discernible clues to the quality of the bonding. Once connected none separated from handling.

### 3.1. Microstructure

**Figure 4:**
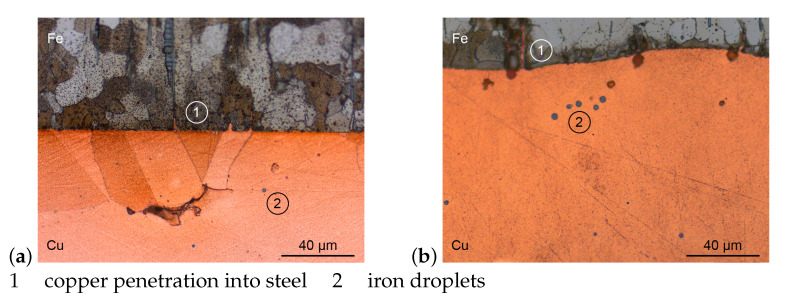
Micrographs of Cu-Fe interface (**a**) 1090 °C for 0 s and (**b**) 1150 °C for 30 s.

Figure 4 shows the interface area of etched Cu-Fe specimens, prepared as described above. As an example, both extrema regarding maximum temperature and dwell time were chosen to be shown here. Both images show largely connected compounds with some micro porosity within the copper. Any bits of surface roughness of the steel specimens are completely filled by copper in both cases. While the steel specimen in Figure 4a still forms a straight line towards the copper, the boundary in image b shows a distinct wave form.

Image a shows two grains of copper, of which one is recrystallised, seeming to form epitaxy with some steel grains. The liquid copper started to penetrate into the steel along grain boundaries up to about 10 μm deep. Within the copper precipitations of iron formed droplets at around 1 μm diameter. The specimen was heated to 1090 °C and immediately cooled down upon reaching maximum temperature.

Image b only shows one grain of the copper part of a specimen, heated to 1150 °C with a dwell time of 30 s. The copper fills any gaps inside the steel, especially along grain boundaries up to around 30 μm deep. Even spaces are filled, which do not show a connection to the copper volume in the image plain, suggesting the liquid copper to meander through the steel. Again, droplets of steel form inside the copper at around 4 μm in diameter.

Both an increase in maximum temperature and dwell time result in increased solution of iron in the liquid copper. The results are an increasing number and size of iron droplets within the copper grains and an increasingly rough interface due to an inhomogeneous diffusion speed.

### 3.2. Hardness

**Figure 5:**
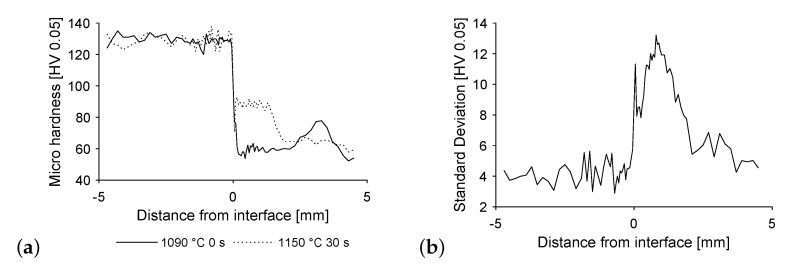
Microhardness (**a**) over the length of the specimen from steel to copper and (**b**) standard deviation of hardness for all temperature-time variations.

Figure 5a shows the microhardness, starting from the open steel face, across the interface up to the free copper face of the specimen. The same specimen are shown as above, namely, those featuring extrema of maximum temperature and dwell time. The hardness values show little fluctuation while within the steel, followed by a sharp drop into the copper. Depending on the extent of steel diffused into the copper, a plateau of hardness values forms at the interface, reaching more or less into the copper. Additionally, a slight increase of hardness where the copper penetrates into the steel is discernible. The hardness values inside the copper are more unsteady, possibly due to as cast structure and segregation effects.

Image b shows standard deviation over all temperature-time variations depending on distance from the interface. This supports the findings of lowest hardness deviations within the steel part of the specimen, followed by the pure copper part. The changing diffusion depth of the steel into the copper creates large deviations in the affected area.

Increasing with maximum temperature and dwell time, the steel migrates further into the copper specimen. This results in elevated hardness values, correlating to the findings above. Yet, hardness is widely unaffected by those parameters, merely diffusion depth increases.

**Figure 6:**
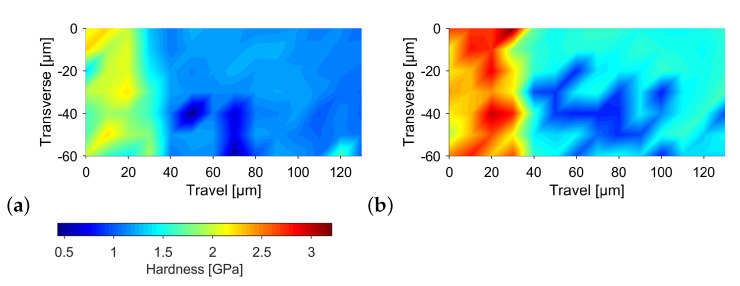
Nanoindentation at the interface (**a**) 1090 °C for 0 s and (**b**) 1150 °C for 30 s.

Figure 6 shows hardness values generated by the nanoindenter. The measuring grid contained 7 by 14 indents equally spaced at 10 μm. The interface can be seen at a longitudinal of approximately 30 μm. Therefore, the first three rows of the grid oriented in transverse direction lie within the steel. Both images show a significant difference of hardness in steel and copper. Image a shows the same specimen as introduced above, produced at a maximum temperature of 1090 °C and without a dwell time. Here, a rather uniform hardness distribution in each zone can be seen, which varies around 2 GPa in steel and around 1 GPa in copper. Image b shows the specimen produced at a maximum temperature of 1150 °C and a dwell time of 30 s. The hardness values are on average around 0.5 GPa higher than those mentioned previously. Only the minima match each other in both images. This leads to the conclusion that the base material, represented by the minima is unaffected by the different temperature-time history. The higher maxima in image b are a result of a higher diffusion activity. As such, both steel and copper have their hardness increased locally, confirming the findings of an increased microhardness within the steel interface.

### 3.3. Mechanical Testing

**Figure 7:**
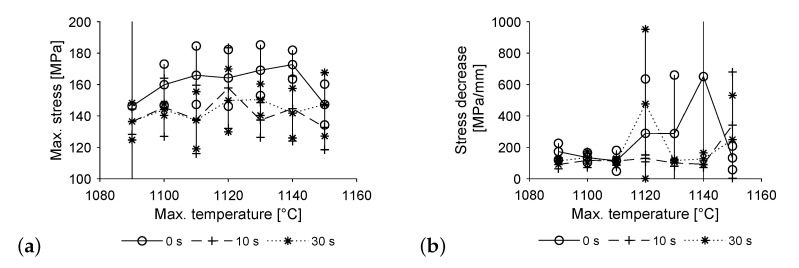
Tensile testing (**a**) maximum stress and (**b**) rate of stress decrease to 70% of maximum stress.

Figure 7 shows the results of the tensile tests, performed on the specimen without a hole. The results are displayed including respective standard deviation for each temperature-time profile, based on 5 specimen each. Image a shows higher values of maximum stress for specimen, which did not have a dwell time at maximum temperature. A plateau forms, starting at approximately 1110 °C. The comparatively low value at 1150 °C matches those of longer dwell times, but seems to be an outlier from the curve, possibly due to increased wear of the mirror furnaces lamps, resulting in a longer heating cycle. The results for both 10 and 30 s resting time are very close to each other and on a rather stable level in average over all temperatures. Only the value at 1090 °C seems a bit lower than the others. Standard deviation is very high for a maximum temperature of 1090 °C and no dwell time, as some of these specimen broke during machining. In this case, the specimen was included in the data assuming it to withstand a maximum stress of 0 MPa. Therefore, a worst case scenario is applied, underestimating their real strength, yet showing the premature failure in the graph.

Image b shows the decrease in stress upon reaching the specimens maximum value. A stress reduction by 30% is divided by the specimens elongation during that time. As such, the values shown represent the failure mode of the specimen, with low values representing ductile behaviour. For all temperatures, at least some specimen show ductile failure where the copper part of the specimen constricts just below its head. At 1090 °C, some of the specimen without a dwell time at maximum temperature break at the copper side of the interface leaving a thin layer of copper on the steel. Those show less ductile breaking and therefore higher values starting at approximately 200 MPa/mm. The rest of the specimen show ductile failure up to 1110 °C, at which point, the ductility of the specimen without a dwell time gradually declines. The specimen with a dwell time of 30 s show ductile failure up to 1140 °C. Yet again, the values at 1150 °C and no dwell time seem to be runaway, as they drop sharply when compared to the previous values. Both resting times show reduced ductility for a maximum temperature of 1050 °C.

**Figure 8:**
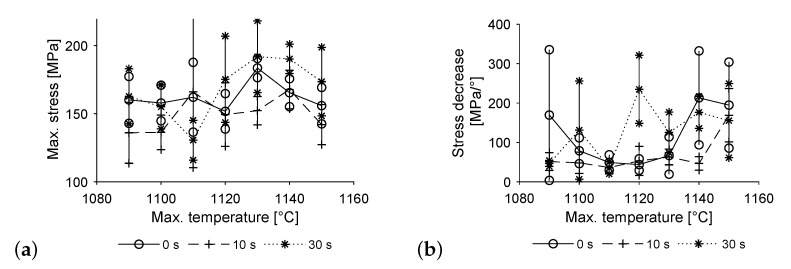
Torsion testing (**a**) maximum stress and (**b**) rate of stress decrease to 70% of maximum stress.

Figure 8 shows the results of the torsion tests. Yet again, the data consists of 5 specimen for each temperature-time profile. Maximum stress values remain relatively constant for a dwell time of 0 s, only showing one outlier at 1130 °C. A dwell time of 30 s results in a pronounced increase of maximum stress at higher temperatures, preceded by a drop in values at 1120 °C. The maximum stress values of a dwell time of 10 s show a slight increase with temperature, dropping only at the highest temperature of 1150 °C.

Again, image b shows the decrease in stress to 70% upon reaching its maximum, divided by the rotation in that time span. The specimens show brittle behaviour in connection with high uncertainty both in the low and in the high temperature ranges when produced without a dwell time at maximum temperature. Specimens produced with dwell times of 10 and 30 s show an increasingly brittle behaviour with higher maximum temperatures overall. Yet again the results for a dwell time of 30 s are rather unsteady. As ductile failure means crack initiation within the pure copper region of the specimens, this is equivalent to the bonding strength exceeding the strength of pure copper. Unfavourable conditions for compound formation prevail at low maximum temperatures and short dwell times, limiting diffusion and wetting effects. Hence it is assumed, that the interface did not reach maximum strength by the time the cooling phase started, resulting in its failure under load. For high temperatures and long dwell times hardening reaches up to 3 mm from the interface into the copper specimen, according to Figure 5b. Therefore, nearly all the testing length is affected, supporting the remaining bit of pure copper under torsional load. Thus, the interface tends to fail.

The results of tensile and torsion tests regarding maximum stress closely match each other; the amount of variation is most likely due to the small specimen size. However, when it comes to failure behaviour, the different states of stress come into play. The differences can be seen most clearly at lower temperatures, resulting in brittle fracture in some cases of the torsion tests where there was rather ductile behaviour in tensile testing. As such, the torsion tests make the transition from ductile to brittle fracture more discernible and measurable, as there is a more densely populated mid-section of stress decreasing values.

## 4. Conclusions

A process could be established, allowing for the stable creation of metallurgically bonded bimetals and their characterisation. The freely choosable temperature-time profile allows for the necessary degrees of freedom to distinguish different modes of action during compound forming. Due to the significant reduction in process times for each specimen, a larger number could be produced when compared to traditional methods and therefore allowing for statistical approaches. Mechanical testing required adaption of standards and testing equipment but proved to produce consistent results. The further testing methods require no alteration. When compared to traditional casting technology, where setup and resulting process boundaries limit the freedom to choose a temperature-time profile, the method presented here offers a rather unique way to analyse interface forming processes as seen in different casting processes ranging from strip casting to melting a cladding material within a base part. Thus, the method supports research activities by providing a way to investigate situations from all kinds of casting processes within one setup. As such, the process could be introduced successfully. Further steps will include an extension to different materials and a thorough comparison between specimen produced by the new method and traditional casting technology.

## Figures and Tables

**Table 1:** Chemical composition of compound components.

Element (mass %)	Cu	Fe	C	Mn	Ni	Cr
Cu-ETP	≥99.9	0.03	-	-	-	-
Steel	0.02	98.3	0.13	1.04	0.02	0.03

## Data Availability

Data sharing is not applicable to this article.

## References

[1] Nerl C., Wimmer M., Hoffmann H., Kaschnitz E., Langbein F., Volk W. (2014). Development of a continuous composite casting process for the production of bilayer aluminium strips. J. Mater. Process. Technol..

[2] Hasse S. (2007). Gießerei Lexikon. 19.

[3] Ißleib A., Friedel A., Lubojanski I. (1995). Verbundgießen von Eisen-Kohlenstoff-Legierungen–grundlegende metallurgische Reaktionen an der Grenzfläche–Teil I. Gießerei-Prax..

[4] Mittler T. (2021). Verbundstranggießen von Kupferwerkstoffen. Ph.D. Thesis.

[5] Steeg M., Engel U., Roemer E. (1993). Hochleistungsfähige metallische Mehrschichtverbundwerkstoffe für Gleitlager. Gleitlager Als Mod. Maschinenelemente. Teil A: Konstr. Werkst. Und Schmier. Von Radiallagern. Expert Ehningen.

[6] Behrens V. (2010). Elektrische Kontakte: Werkstoffe, Gestaltungen und Anwendungen in der Nachrichten-, Automobil-und Energietechnik.

[7] Flores V., Almanza R. (2004). Behavior of the compound wall copper–steel receiver with stratified two-phase flow regimen in transient states when solar irradiance is arriving on one side of receiver. Sol. Energy.

[8] Hans M., Támara J.C., Mathews S., Bax B., Hegetschweiler A., Kautenburger R., Solioz M., Mücklich F. (2014). Laser cladding of stainless steel with a copper–silver alloy to generate surfaces of high antimicrobial activity. Appl. Surf. Sci..

[9] D’Andrea D., Epasto G., Bonanno A., Guglielmino E., Benazzi G. (2019). Failure analysis of anti-friction coating for cylinder blocks in axial piston pumps. Eng. Fail. Anal..

[10] Abbasi M., Toroghinejad M.R. (2010). Effects of processing parameters on the bond strength of Cu/Cu roll-bonded strips. J. Mater. Process. Technol..

[11] Liu H., Zhang B., Zhang G. (2011). Enhanced toughness and fatigue strength of cold roll bonded Cu/Cu laminated composites with mechanical contrast. Scr. Mater..

[12] Vinaricky E. (2002). Elektrische Kontakte, Werkstoffe und Anwendungen.

[13] Pan D., Gao K., Yu J. (1989). Cold roll bonding of bimetallic sheets and strips. Mater. Sci. Technol..

[14] Münster D., Hirt G. (2019). Copper Clad Steel Strips Produced by a Modified Twin-Roll Casting Process. Metals.

[15] Chen G., Xu G. (2017). Effects of melt pressure on process stability and bonding strength of twin-roll cast steel/aluminum clad sheet. J. Manuf. Process..

[16] Kadoshnikov V.I., Kulikova E.V., Dema R.R., Kharchenko M.V., Androsenko M.V., Latypov O.R. (2019). Manufacturing Technology Improvement of Technology and Equipment for Preparing Steel-Copper Wire. Chem. Pet. Eng..

[17] Kasper W. (2000). Dynaplat^®^ Sprengplattierung-eine explosive Verbindung. STAHL.

[18] Langeslag S., Sgobba S., Libeyre P., Gung C.Y. (2015). Extensive characterisation of copper-clad plates, bonded by the explosive technique, for ITER electrical joints. Phys. Procedia.

[19] Haubenberger W. (1980). Ein Kupfer-Austenit-Verbundleiter fuer hohe mechanische Beanspruchung. Metall.

[20] Zhi-shui Y., Feng-jiang W., Xiao-quan L., Ming-fang W. (2000). Diffusion bonding of copper alloy to stainless steel with Ni and Cu interlayer. Trans. Nonferrous Met-Als Soc. China.

[21] Dong L., Chen W., Hou L., Liu Y., Luo Q. (2016). Metallurgical process analysis and microstructure characterization of the bonding interface of QAl9-4 aluminum bronze and 304 stainless steel composite materials. J. Mater. Process. Technol..

[22] Sürül K. (2009). Entwicklung von neuartigen Bindeschichten zur Verbesserung der Bindefestigkeit von Zinnbasis-Gleitlagermetallen.

[23] Hejazi M.M., Divandari M., Taghaddos E. (2009). Effect of copper insert on the microstructure of gray iron produced via lost foam casting. Mater. Des..

[24] (2018). DIN EN ISO 7500-1 Metallic Materials-Calibration and Verification of Static Uniaxial Testing Machines-Part 1: Tension/Compression Testing Machines-Calibration and Verification of the Force-Measuring System.

[25] (2020). DIN EN ISO 6892-1:2020-06, Metallische Werkstoffe-Zugversuch-Teil 1: Prüfverfahren bei Raumtemperatur (ISO 6892-1:2019).

